# Tracing the transmission dynamics of HIV-1 CRF55_01B

**DOI:** 10.1038/s41598-020-61870-x

**Published:** 2020-03-20

**Authors:** Junjie Zai, Haizhou Liu, Zhenzhen Lu, Antoine Chaillon, Davey Smith, Yi Li, Xingguang Li

**Affiliations:** 1 Hubei Engineering Research Center of Viral Vector, Wuhan University of Bioengineering, Wuhan, 430415 China; 2 Centre for Emerging Infectious Diseases, The State Key Laboratory of Virology, Wuhan Institute of Virology, University of Chinese Academy of Sciences, Wuhan, 430071 China; 3 Non-communicable Chronic Disease Prevention & Control Division, Guangxi Center for Disease Control and Prevention, Nanning, 530028 China; 4 Department of Medicine, University of California San Diego, California, United States of America

**Keywords:** Genome informatics, Evolutionary genetics

## Abstract

To investigate the genetic diversity, spatiotemporal dynamics, and transmission networks of HIV-1 CRF55_01B epidemic in China. A total of 209 partial *pol* gene sequences of HIV-1 CRF55_01B were sampled during 2007–2015 from 7 provinces of China. Phylogenetic analyses and trait diffusion process of these sequences were performed using Bayesian methods. Distance-based molecular network analyses were performed to infer putative relationships. Characteristics of genetically linked individuals were analyzed. Our study identified that HIV-1 CRF55_01B likely originated among men who have sex with men (MSM) in Guangdong province in January 2003 (April 2000–April 2005), and that Guangdong province and MSM are major hubs for the spread of the HIV-1 CRF55_01B epidemic in China. A Bayesian Skygrid plot revealed that the effective population size of HIV-1 CRF55_01B experienced increased phase followed by a plateau. All sequences from persons of unknown risk clustered within groups who reported MSM risk. This could be because Chinese MSM may not report such risk due to HIV/AIDS-related stigmatization and discrimination. This study inferred the transmission dynamics of the HIV-1 CRF55_01B epidemic in China at high resolution. The methods developed in this study may be critical for designing effective HIV prevention strategies in China and beyond.

## Introduction

The prevalence of HIV-1 among men who have sex with men (MSM) continues to increase in China, especially in its big cities, like Beijing, Shanghai, Guangzhou, Shenzhen, Shenyang, Shijiazhuang^1,2^. The main HIV-1 subtypes circulating in China are subtype B’ and circulating recombinant forms (CRFs), including CRF01_AE, CRF07_BC, CRF08_BC^3^. Co-circulation of multiple subtypes of HIV-1 strains among high risk groups, like injecting drug users (IDUs) and MSM, favors the generation of new CRFs^4,5^. Interestingly, CRF 55_01B was first reported in 2013 among MSM from Changsha city of Hunan province and Dongguan city of Guangdong province in China and it was composed of CRF01_AE and subtype B^6^. However, the earliest known strain of CRF55_01B was traced back to 2007 in sample from Shenzhen of Guangdong province among MSM^7^. Now, CRF55_01B is mainly distributed in Guangdong and neighboring provinces in China, and is found across all risk groups^7,8^. Despite this research, we still have an incomplete understanding the origin and evolutionary history of the CRF55_01B epidemic in China.

In the present study, we employed state-of-the-art methods to define the spatiotemporal dynamics, transmission networks, geographic origins, and migration patterns of CRF55_01B based on 209 partial *pol* gene sequences of CRF55_01B with known sampling dates (2007–2015) and geographic locations (7 provinces) primarily among MSM in China. We also estimated the maximum posterior probability risk group for each sequence with unknown risk, for the first time, to uncover the key socio-cultural factors (e.g. HIV/AIDS-related stigmatization and discrimination) behind it. Our study provides insights into the origin and evolutionary history of CRF55_01B epidemic in China. The methods developed in this study may be critical for designing effective HIV prevention strategies in China and beyond.

## Materials and Methods

### Sequence data set

All available partial *pol* gene sequences of CRF55_01B (2253–3308 nt relative to HXB2) with known sampling year and province of collection were retrieved from the Los Alamos National Laboratory (LANL) HIV Sequence Database (http://www.hiv.lanl.gov). Where multiple sequences were available per individual, only one was selected. Quality Control and RIP v.3.0^9^ from the LANL site were used to analyze the quality and confirm the genotype assignment of all sequences, respectively. Hypermut v2.0 from the LANL site was performed to analysis the hypermutation of all sequences^10^. For this data set, sequences were aligned using Gene Cutter from the LANL site and then adjusted manually using BioEdit v7.2.5^11^. The final dataset included 209 partial *pol* gene sequences of CRF55_01B with known sampling year and province between 2007–2015.

### Phylogenetic analyses

To examine the phylogenetic signal for this data set, a likelihood-mapping analysis^12^ was performed using TREE-PUZZLE v5.3.rc16^13^. To evaluate the temporal structure for this data set, we performed root-to-tip genetic distance against year of sampling using TempEst v1.5^14^. We then employed a Bayesian phylogenetic approach to estimate the rate of evolution and the time to the most recent common ancestor (tMRCA) for this data using a GTR + G substitution model with an uncorrelated lognormal relaxed-clock model^15^ and a Bayesian Skygrid coalescent tree prior^16^ in BEAST v1.8.2^17^. The Markov chain Monte Carlo (MCMC) analysis was run for 500 million steps with sampling every 50,000 steps. Convergence was evaluated by calculating the effective sample sizes (ESSs) of the parameters using Tracer v1.7.1^18^. Trees were summarized as maximum clade credibility (MCC) trees using TreeAnnotator after discarding the first 10% as burn-in, and then visualized in FigTree v1.4.3 (http://tree.bio.ed.ac.uk/software/figtree).

To test the hypothesis that a tip with a province or risk group is more likely to share that discrete-trait with a neighboring adjoining tip than would be expected by chance, we calculated the association index (AI), Fitch parsimony score (PS), and monophyletic clade size (MC) statistics for each discrete-trait using Bayesian Tip-Significance Testing (BaTS) v0.9 beta^19^, as previous described^20^. We reject the null hypothesis for a significance level of 0.001, 0.001, and 0.05 for AI, PS, and MC statistics, respectively.

### Ancestral reconstructions of discrete traits

We employed a Bayesian phylogenetic method to infer the ancestral discrete traits for this data set. To do this, we modelled two types of traits (e.g. geographic location and risk group) as a diffusion process among discrete states^21^ in BEAST v1.8.2^17^. Diffusion among discrete traits was modelled using a non-reversible continuous-time Markov chain^21^. Bayesian stochastic search variable selection was used to identify non-zero migration rates between each pair of traits. In addition, we estimate the expected number of viral migrations using ‘Markov jump counts’ approaches^22–24^ between each pair of traits. The phylogeographic history was visualized using SpreaD3 v0.9.6^25^.

### Transmission network reconstruction

We employed HIV TRAnsmission Cluster Engine (HIV-TRACE; www.hivtrace.org)^26^ to infer transmission network clusters for this data set (e.g. closely related sequences inferring a transmission network). All pairwise distances were calculated and a putative linkage between each pair of two sequences was considered whenever their divergent was ≤0.02 substitutions/site (TN93 substitution model). When calculating pairwise genetic distance, all nucleotide ambiguities were resolved and only sequences with less than 0.2% ambiguities were retained. Multiple linkages were then combined into putative transmission clusters. Clusters comprised of only two linked nodes were identified as dyads. This approach detected clusters of recent transmission in which the clustering viruses are genetically similar, implying a direct or indirect epidemiological connection^27^.

## Results

### Social-demographic characteristics of this data set

This data set included 209 sequences of CRF55_01B strains from various risk groups: heterosexuals (Hetero, *n* = 27), injecting drug users (IDUs, *n* = 1), MSM (*n* = 169), and unknown risk (n/a, *n* = 12). The samples are obtained from 7 Chinese provinces: Anhui (*n* = 2), Guangdong (*n* = 166), Guangxi (*n* = 8), Hebei (*n* = 10), Hunan (*n* = 2), Shanghai (*n* = 18), and Zhejiang (*n* = 3), with sampling years between 2007 and 2015 (Table 1 and Supplementary Table S1). The main risk groups were MSM (80.86%) and Hetero (12.92%). The samples were primarily from Guangdong (79.43%) and Shanghai (8.6%).

**Table 1:** Geographic source, sampling year, and risk factor for HIV-1 CRF55_01B strains used in the present study.

Geographic source	Sampling year	n	Risk factor^a^			
			Hetero	IDU	MSM	n/a
Anhui	2011	2			2	
Guangdong	2007–2013	166	23	1	142	
Guangxi	2009–2013	8	1			7
Hebei	2013–2015	10	2		4	4
Hunan	2010–2011	2			2	
Shanghai	2011–2013	18			18	
Zhejiang	2013–2014	3	1		1	1
**Total**		**209**	**27**	**1**	**169**	**12**

^a^Risk factor: Hetero, heterosexual; IDU, injecting drug user; MSM, men having sex with men; n/a, not available.

### Transmission network analysis

**Figure 1:**
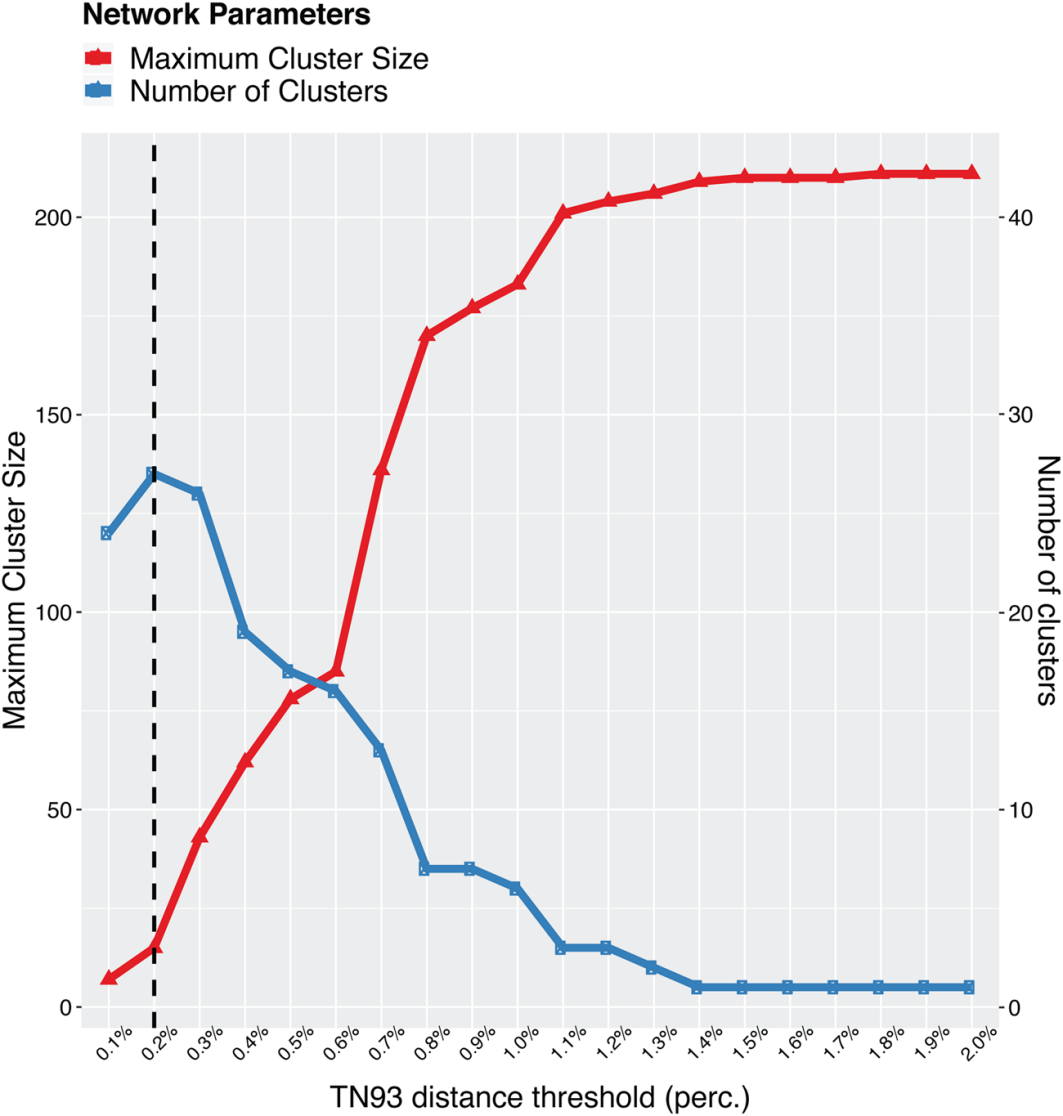
Number of clusters and maximum cluster size, as a function of the TN93 distance threshold. Genetic distance at 0.002 substitutions/site was highlighted with a dash line. Color-coded network parameters are shown on the top left.

**Figure 2:**
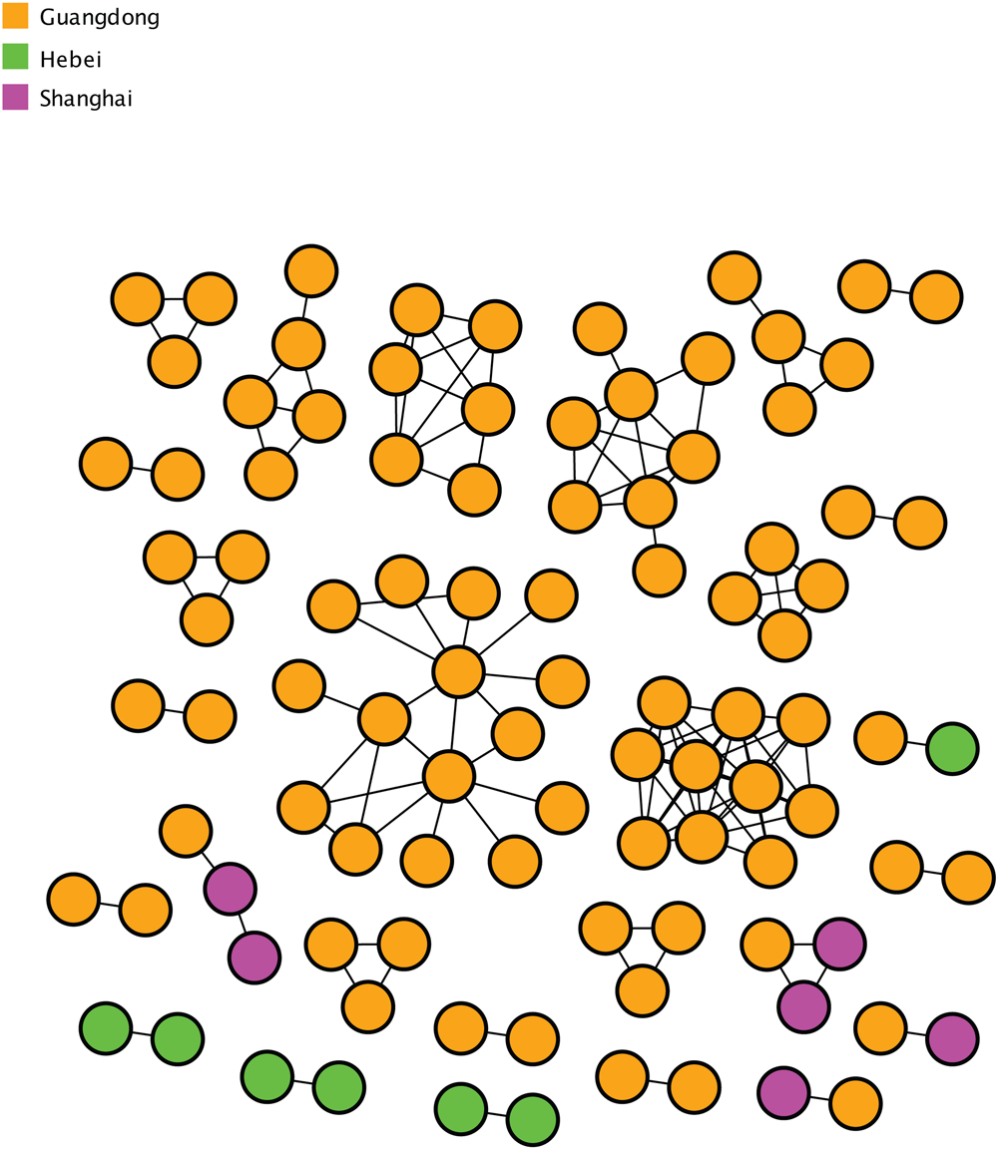
Transmission clusters of HIV-1 CRF55_01B. The structure of inferred CRF55_01B transmission clusters from our data set are illustrated. Nodes (circles) represent connected individuals in the overall network, and putative transmission linkages are represented by edges (lines). Nodes are color coded by the province of origin.

To identify putative genetic linkage between 2 individuals, we first performed a sensitivity analysis across a plausible range of genetic distance threshold ranging from 0.1% to 2.0%. We found that transmission clusters started to coalesce and the transmission network lost resolution when the genetic distance >0.2% for this data set (Fig. 1). Therefore, we considered individuals as being genetically linked when the genetic distance between HIV-1 CRF55_01B *pol* sequences was <0.2%. This allowed us identify 27 clusters that included 98/209 (46.89%) sequences. Clusters ranged in size from 2 to 15 sequences, and 13 (48.15%) had 3 or more sequences (Fig. 2; Supplementary Fig. S1; Supplementary Table S2 and S3). Two clusters included at least 10 sequences, and were considered ‘large’ clusters. Both of the two large clusters included sequences exclusively from Guangdong, ranging in size from 10 to 15 sequences (Fig. 2; Supplementary Table S2 and S3). The largest cluster of 15 sequences included exclusively from MSM. However, the other large cluster of 10 sequences included both from MSM and Hetero, and was a predominantly comprised of sequences from people with ‘Hetero’ risk (Supplementary Fig. S1; Supplementary Table S2 and S3).

Of the 27 inferred clusters, 84 (85.71%), 7 (7.14%), and 6 (6.12%) included individuals sampled in Guangdong, Hebei, and Shanghai. Notably, 19 (70.37%) and 3 (11.11%) of 27 clusters were comprised of individuals from only Guangdong and Hebei, respectively. 5 (18.52%) of 27 clusters included sequences from two provinces: 4 clusters included sequences from Guangdong and Shanghai, and 1 from Guangdong and Hebei (Fig. 2; Supplementary Table S2 and S3).

We found that 6 clusters (22.22%) included persons reporting both Hetero and MSM, and only 1 cluster (3.70%) included persons only reporting Hetero risk (Supplementary Fig. S1; Supplementary Table S2 and S3).

Of the 27 (12.92%) sequences from individuals identifying as Hetero, 14 (51.85%) clustered with at least one other sequence, and 83 (49.11%) of the 169 sequences from individuals identifying as MSM clustered.

### Likelihood-mapping and evolutionary divergence analysis

For this data set, our likelihood-mapping analysis revealed a strong phylogenetic signal (Supplementary Fig. S2). The correlation between root-to-tip distances and sampling year indicated a relative strong temporal signal (*R*^2^ = 0.307), with an estimated substitution rate of 1.64 × 10^−3^ substitutions per site per year and the time to the most recent common ancestor of July 2003 (Supplementary Fig. S3).

Bayesian evolutionary analyses revealed that the evolutionary rate of CRF55_01B was 1.68 × 10^−3^ substitutions per site per year [95% highest posterior density (HPD) interval: 1.33 × 10^−3^–2.05 × 10^−3^), and tMRCA of CRF55_01B was January 2003 (95% HPD interval: April 2000 and April 2005).

**Figure 3:**
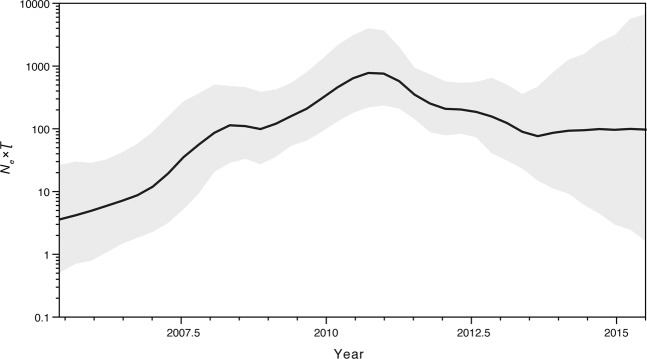
Bayesian Skygrid demographic reconstruction of HIV-1 CRF55_01B. The vertical axis shows the effective number of infections (*N*_*e*_) multiplied by mean viral generation time (τ). The solid line and shaded region represent the median and 95% credibility interval, respectively, of the inferred *N*_*e*_τ through time.

We further investigated the estimated population dynamics of CRF55_01B using a Bayesian Skygrid plot, which reflects the changes in effective population size (*Ne*) over time. The dynamic of the *Ne* showed two distinct phases: (1) exponential growth (2005–2008 and 2008–2010) followed by (2) declining phase (2011–2013) (Fig. 3).

### Phylogenetic association with geographic location and risk group

Based on the AI and PS statistics of geographic location and risk group traits, we rejected the null hypothesis with *P* < 0.001 (Tables 2 and 3). For the MC statistic of geographic location trait, we also rejected the null hypothesis (*P* < 0.05), with the exception of the MC (Anhui) and MC (Shanghai) statistics (Table 2). However, for the MC statistic of risk group trait, we accepted the null hypothesis (*P* > 0.05) (Table 3). Both the transmission network and MC statistic model analyses showed mixed between risk group (e.g. MSM and Hetero).

**Table 2:** Statistical analysis of province of CRF55_01B sequences used in the present study.

Statistic	No. of sequences	Observed mean (95% CI)	Null mean (95% CI)	*P*-value
AI		5.2 (4.5, 5.8)	8.4 (7.4, 9.4)	0*
PS		31.9 (30.0, 33.0)	42.1 (40.7, 42.9)	0*
MC (Anhui)	2	1.0 (1.0, 1.0)	1.0 (1.0, 1.0)	N/A
MC (Guangdong)	166	15.3 (14.0, 17.0)	10.6 (8.2, 14.8)	0.0356*
MC (Guangxi)	8	2.0 (2.0, 2.0)	1.1 (1.0, 1.8)	0.0263*
MC (Hebei)	10	2.0 (2.0, 2.0)	1.1 (1.0, 2.0)	0.0484*
MC (Hunan)	2	2.0 (2.0, 2.0)	1.0 (1.0, 1.0)	0.0011*
MC (Shanghai)	18	2.0 (2.0, 2.0)	1.4 (1.0, 2.0)	0.1724
MC (Zhejiang)	3	2.0 (2.0, 2.0)	1.0 (1.0, 1.0)	0.0028*

AI, association index.

PS, parsimony score.

MC, monophyletic clade statistic.

95% CI, 95% credbility interval.

*Statistically significant (P < 0.05).

N/A, not available because of the observed 95% CI contains the null 95% CI.

**Table 3:** Statistical analysis of risk group of CRF55_01B sequences used in the present study.

Statistic^a^	No. of sequences	Observed mean (95% CI)	Null mean (95% CI)	*P*-value
AI		5.2 (4.3, 6.2)	7.6 (6.6, 8.6)	0*
PS		32.5 (30.0, 35.0)	38.4 (36.6, 39.7)	0.0001*
MC (Hetero)	27	2.4 (2.0, 4.0)	1.8 (1.1, 2.3)	0.4126
MC (IDU)	1	1.0 (1.0, 1.0)	1.0 (1.0, 1.0)	N/A
MC (MSM)	169	13.0 (9.0, 18.0)	11.3 (8.7, 15.2)	0.2809
MC (n/a)	12	2.0 (2.0, 2.0)	1.2 (1.0, 2.0)	0.0700

AI, association index.

PS, parsimony score.

MC, monophyletic clade statistic.

95% CI, 95% credbility interval.

*Statistically significant (P < 0.05).

N/A, not available because of the observed 95% CI contains the null 95% CI.

^a^Risk factor: Hetero, heterosexual; IDU, injecting drug user; MSM, men having sex with men; n/a, not available.

### Dynamics analysis of ancestral discrete traits

**Figure 4:**
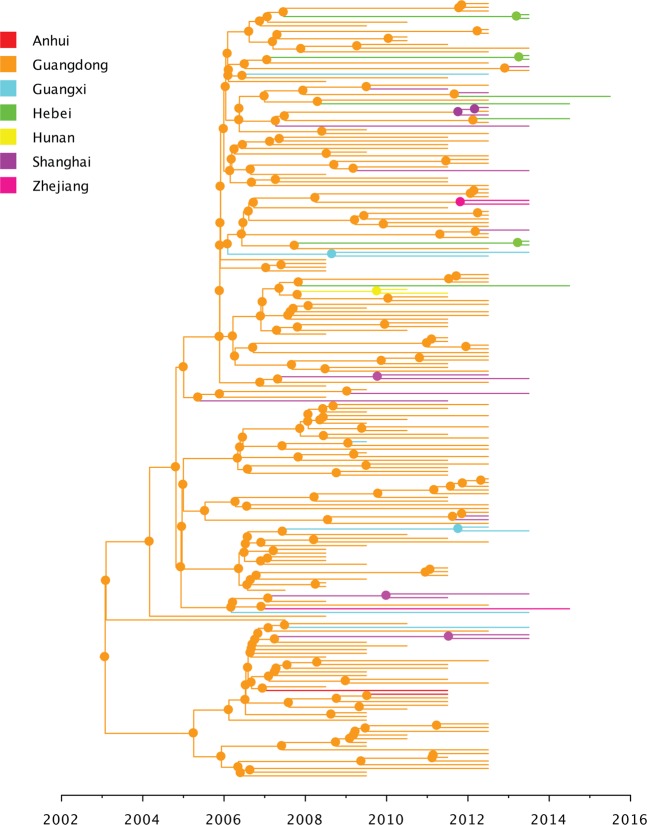
Maximum-clade-credibility tree estimated from partial *pol* gene sequences of HIV-1 CRF55_01B. Nodes are color coded by the most probable geographic location of the descendent branches.

**Figure 5:**
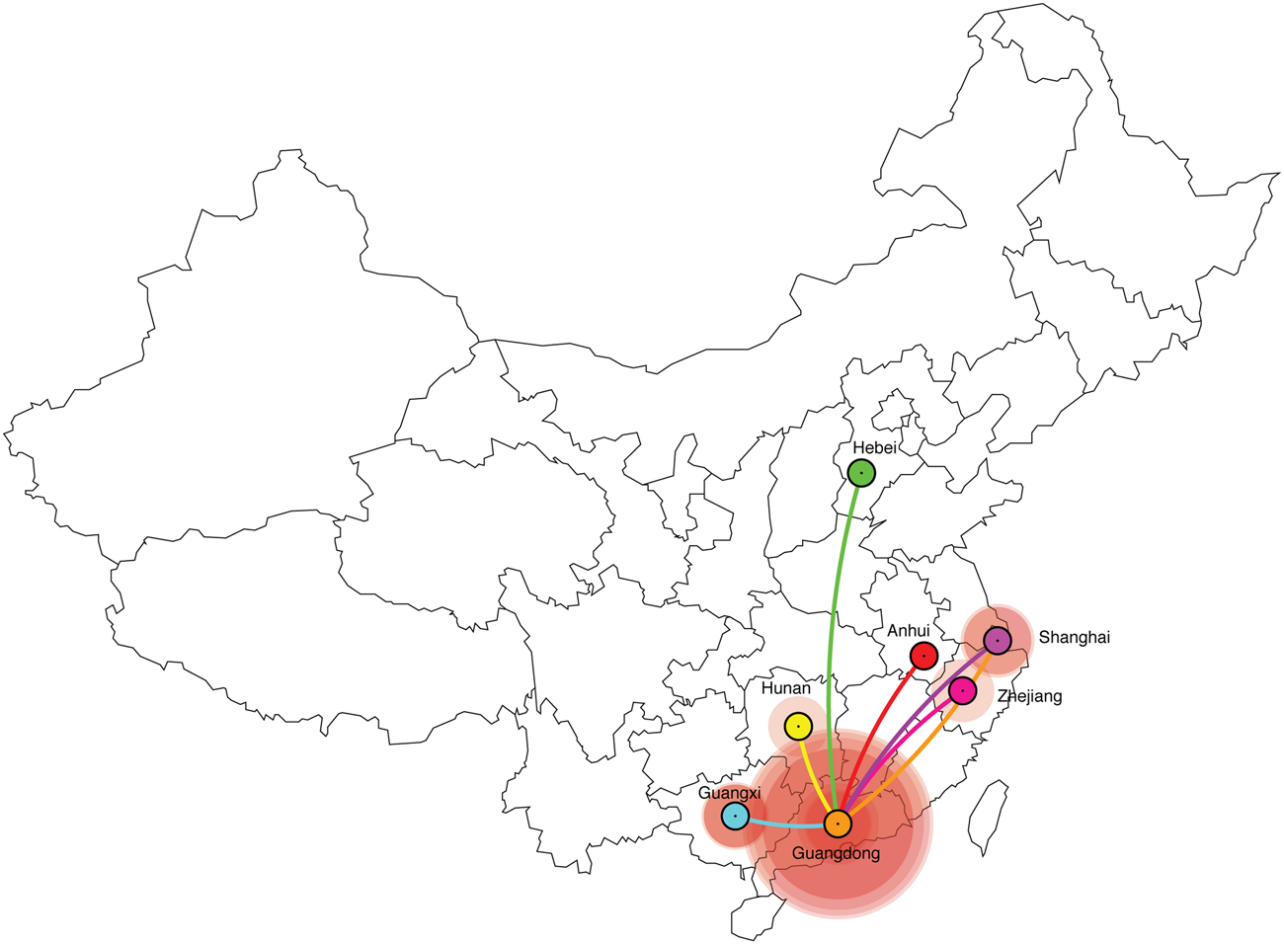
Visualization of geographic location transitions for HIV-1 CRF55_01B overlaid on a geographical map. Points are color coded by the geographic location of origin. Lines are color coded by the geographic location of destination.

Our phylogeographic analysis revealed that the most probable root location of CRF55_01B ancestor was in Guangdong among MSM in January 2003 (posterior state probability = 1.0) (Fig. 4; Supplementary Fig. S4). Our results also revealed that Guangdong acted as a diffusion center to other provinces (Fig. 5). After the introduction of the virus from Guangdong to Shanghai, Shanghai acted as a secondary diffusion province. In addition, there were one bidirectional transition event from the Shanghai to Guangdong (Fig. 5). Most viral transitions between epidemiologically linked provinces were from Guangdong to Shanghai (mean estimate 14.81; 95% HPD interval: 11.70–17.67; Supplementary Fig. S5). Our results also showed that the estimated of the most posterior probability of all of 12 sequences with unknown risk were grouped into MSM ranging from 0.67 to 0.93.

## Discussion

Our evolutionary analyses, based on 209 partial *pol* gene sequences of HIV-1 CRF55_01B that included province of origin and year of sampling, confirmed that this subtype had spread widely within China (Supplementary Table S1)^7,8,28^. Our estimates of the evolutionary rate for CRF55_01B was reassuringly consistent across different methods and models. Further, our estimate of the 95% HPD interval of the evolutionary rate for CRF55_01B (1.33 × 10^−3^–2.05 × 10^−3^) had a very narrow range and also included in those obtained from previous study^7^. Our estimate of the 95% HPD interval of tMRCA for CRF55_01B (April 2000–April 2005) was overlapping with, but not similar to, the reported estimates from two previously studies, which ranged from February 1996 to June 2004^7^ and from June 1996 to January 2004^8^. The uncertainty in our estimate is much smaller than those of previous estimates because of the wider temporal span and more provinces of the samples included in our data set.

Coalescent-based demographic inference revealed a rapidly increasing population size for CRF55_01B from initial phase during 2005–2010, consistent with the outbreak during this period of time. However, we also detected a subsequent phase of rapid declining population size during 2011–2013 (Fig. 3). Furthermore, we found that province was indeed having a significant influence on the complex transmission dynamics of CRF55_01B (Table 2). Phylogeny of CRF55_01B was more likely structured by discrete geographic location traits, especially for Guangdong and Hebei provinces. However, phylogeny of CRF55_01B did not structure by risk group traits, especially for MSM and Hetero risk groups, indicating that MSM and Hetero were mixing with each other (Table 3). This may be explained by that many men in China who reported heterosexual exposure were actually bisexual with ongoing risk exposures to men and women, but they did not report their male sexual contact due to HIV/AIDS-related stigmatization and discrimination^7^. Our results showed that all sequences from persons of unknown risk clustered within groups who reported MSM risk. This could be because Chinese MSM may not report such risk due to HIV/AIDS-related stigmatization and discrimination.

Our phylogeographic reconstruction for this data set demonstrated that the origin of CRF55_01B was located among MSM in Guangdong between April 2000 and April 2005 (Fig. 4; Supplementary Fig. S4). However, we found that Guangdong was the most important hub of dissemination of CRF55_01B outbreak, fueling the origin of new local epidemics in Shanghai and other provinces (Fig. 5). We also identified one bidirectional transition event between Shanghai and Guangdong. These results indicate the complex spatial dynamics of CRF55_01B.

In the present study, we identified 27 highly related clusters within the CRF55_01B outbreak, including two large clusters detected among MSM and heterosexual men and women in Guangdong (Fig. 2; Supplementary Fig. S1; Supplementary Tables S2 and S3). Among the 27 highly related clusters, 22 included sequences from a single province (19 from Guangdong and 3 from Hebei). These clusters ranged in size from 2 to 15 sequences, and included sequences from persons with MSM and heterosexual risk. The other 5 clusters included sequences from two provinces (e.g. Guangdong and Hebei/Shanghai) exclusively from persons with MSM risk. These clusters ranged in size from 2 to 3 sequences. We acknowledge that large clusters are theoretically more likely to include sequences from multiple origins and that these larger clusters may have been identified because of a more intense sampling in a specific region or during a specific period. These results supported a complex scenario of CRF55_01B that was introduced into epidemiologically linked, high-risk groups in China. Such knowledge can be used to help identify new outbreaks of HIV or specific CRFs in near real-time. Since HIV-1 CRF55_01B is primarily exclusive to China, sequences that did not cluster likely represent missed links from inadequate sampling, which is an issue for all molecular epidemiology studies using real world data. Sequences without these links could not be assessed for their origin, therefore we cannot exclude that the possibility that sampling depth hindered our network inferences. Further deep and wide sampling might reveal the presence of additional CRF55_01B clusters. As more sequences are characterized within other provinces of China, more local, regional, and national clusters are likely to emerge, presenting a challenge to HIV control.

Taken together, our results shed light on the spatial and temporal origins of HIV-1 CRF55_01B, and its mode of spread across comparable geographic areas, and suggest that socio-cultural factors (e.g. HIV/AIDS-related stigmatization and discrimination) should attract enough attention in China and beyond. People’s attitudes and practices concerning HIV/AIDS have an important influence on prevention and control HIV spread. Our results also emphasize the importance of using phylodynamic analyses and transmission networks to provide insights into the role of MSM in the spread of HIV in China. These efforts, combined with epidemiological investigation, are needed to track changes in HIV epidemics. Understanding these epidemic dynamics in real time is increasingly public health importance in terms of guiding prevention efforts.

## Supplementary information

SUPPLEMENTARY INFO.
