# Supplementary material for: Tracing the transmission dynamics of HIV-1 CRF55_01B
Source: Sci Rep. 2020 Mar 20;10:5098. doi: 10.1038/s41598-020-61870-x (PMC7083841; doi:10.1038/s41598-020-61870-x)
Supplement: Supplementary file 1 — SUPPLEMENTARY INFO. [file 41598_2020_61870_MOESM1_ESM.pdf]

Tracing the transmission dynamics of HIV-1 CRF55_01B

Junjie Zai[1, *], Haizhou Liu[2, *], Zhenzhen Lu[3, *], Antoine Chaillon[4], Davey Smith[4], Yi Li[1, #] and Xingguang Li [1, *, #]

1. Hubei Engineering Research Center of Viral Vector, Wuhan University of Bioengineering, Wuhan, 430415, China.
2. Centre for Emerging Infectious Diseases, The State Key Laboratory of Virology, Wuhan Institute of Virology, University of Chinese Academy of Sciences, Wuhan, 430071, China.
3. Non-communicable Chronic Disease Prevention & Control Division, Guangxi Center for Disease Control and Prevention, Nanning, 530028, China.
4. Department of Medicine, University of California San Diego, La Jolla, California, United States of America.

Correspondence to:

Dr. Xingguang Li, Hubei Engineering Research Center of Viral Vector, Wuhan University of Bioengineering, Wuhan, 430415, China. Tel: +86-027-89648139, E-mail: xingguanglee@hotmail.com.

Prof. Yi Li, Hubei Engineering Research Center of Viral Vector, Wuhan University of Bioengineering, Wuhan, 430415, China. Tel: +86-027-89648361, E-mail: liyi@whsw.edu.cn.

*These authors contributed equally to this work.

[#]These authors contributed equally to this work.

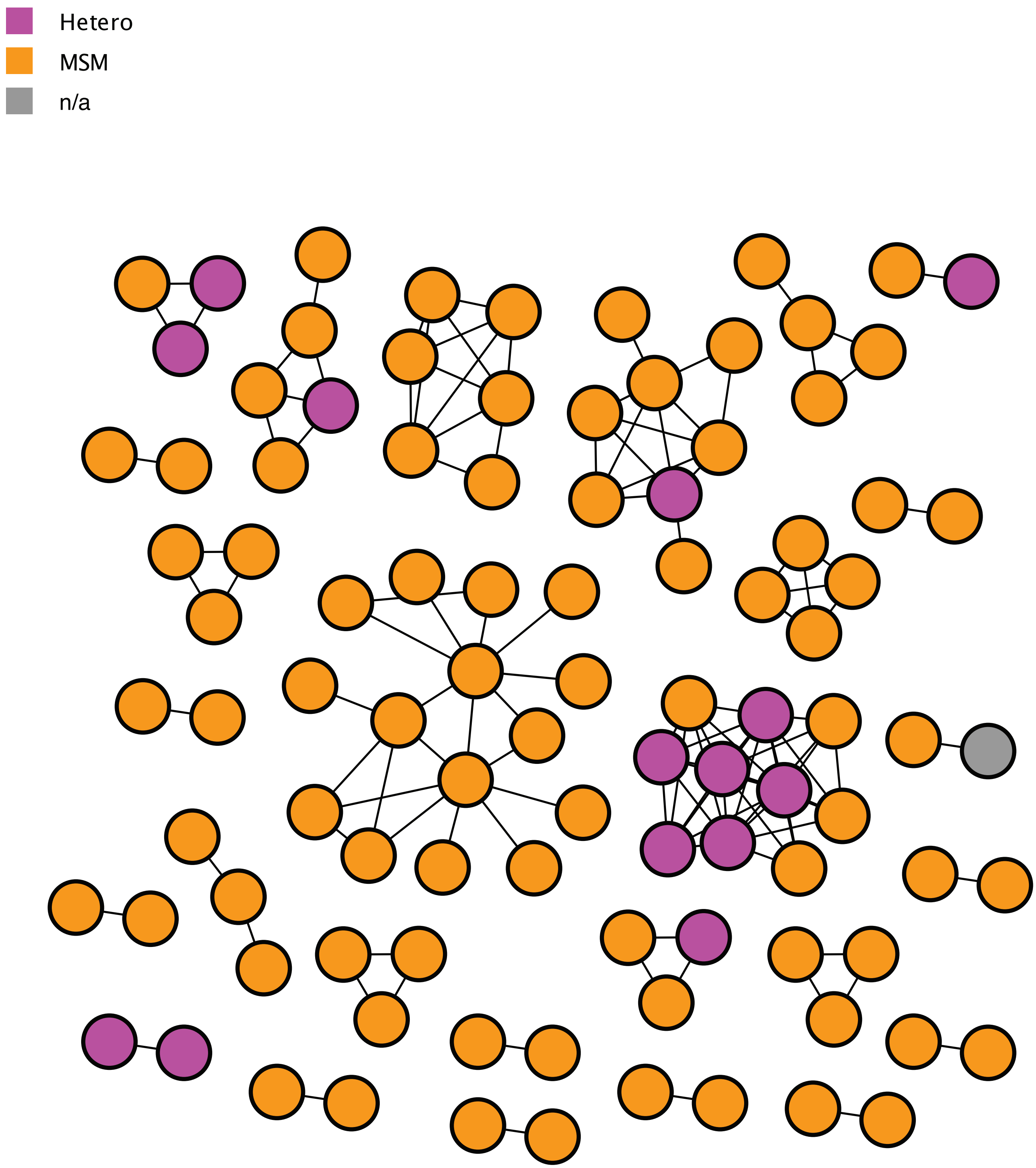

Supplementary Figure S1. Transmission clusters of HIV-1 CRF55_01B.
The structure of inferred CRF55_01B transmission clusters from our data set are illustrated. Nodes (circles) represent connected individuals in the overall network, and putative transmission linkages are represented by edges (lines). Nodes are color coded by the risk group of origin.

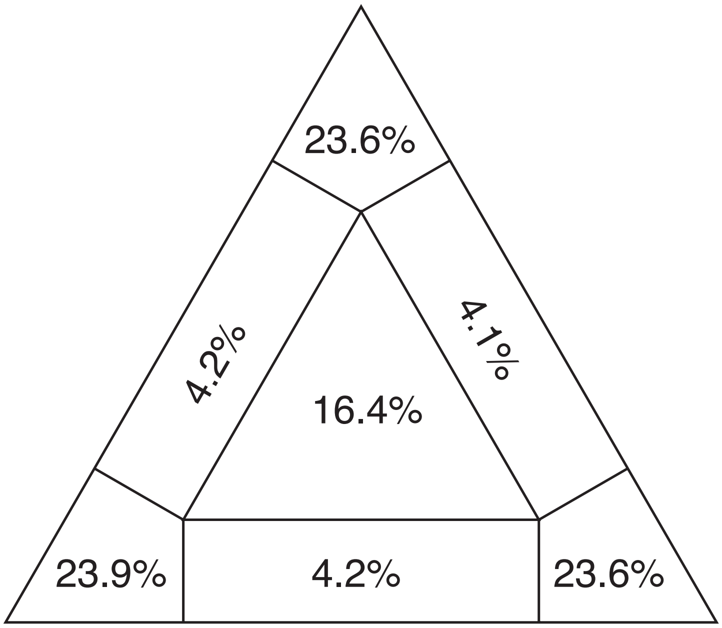

Supplementary Figure S2. Likelihood-mapping analyses of partial pol gene sequences of HIV-1 CRF55_01B.

The likelihoods of the three tree topologies of each possible quartet (or of a random sample of quartets) are denoted by a data point in an equilateral triangle. The distribution of points in the seven areas of the triangle reflects the tree-likeness of the data. Specifically, the three corners represent fully resolved tree topologies; the center represents an unresolved (star) phylogeny; and the sides represent support for conflicting tree topologies.

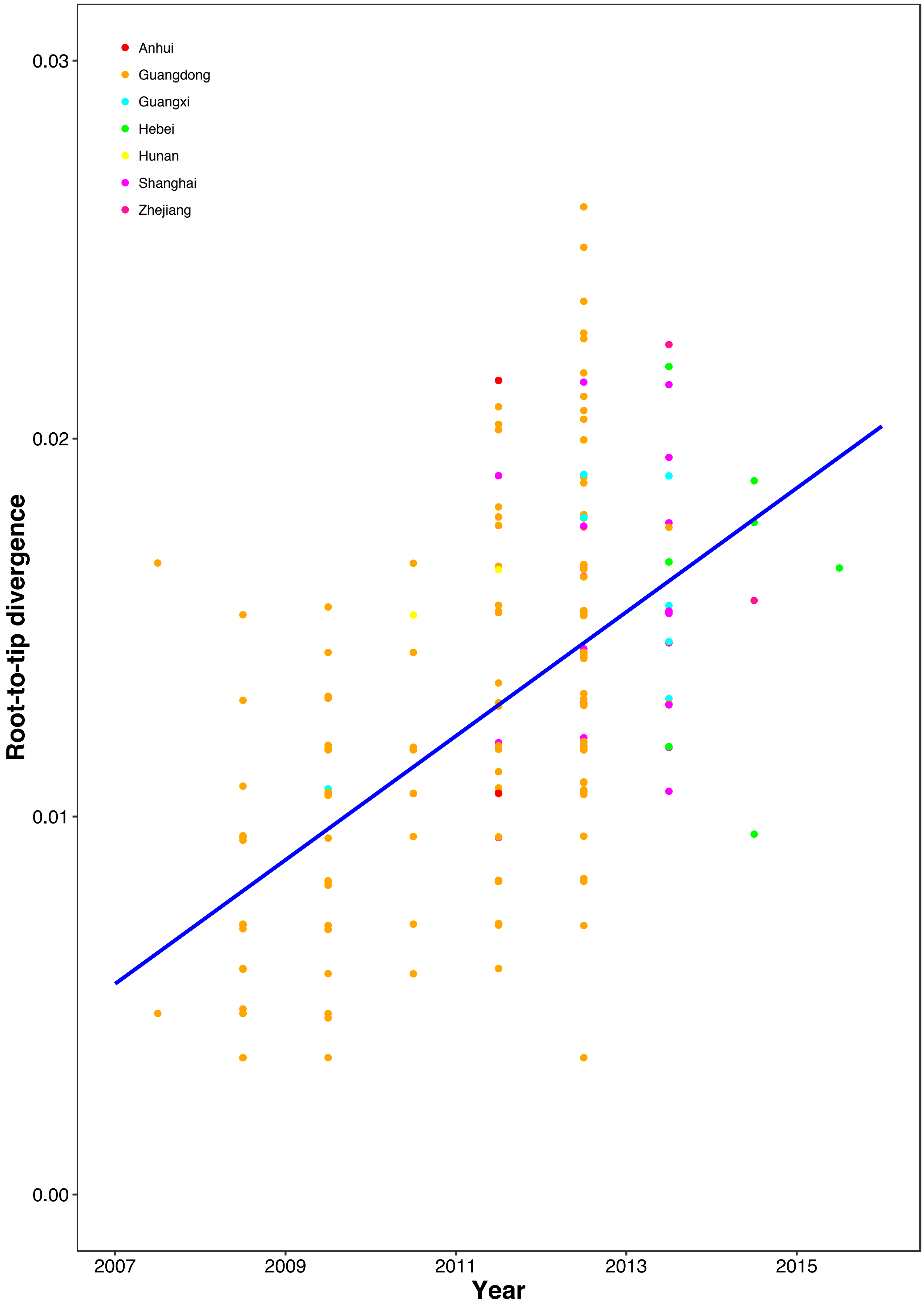

Supplementary Figure S3. Regression of the root-to-tip genetic distance against year of sampling for the HIV-1 CRF55_01B.

Points are color coded by the province of origin. Blue indicates linear regression line. For this study: slope = 0.00164 substitutions per site per year, R^2 = 0.307. Color-coded locations are shown on the top left.

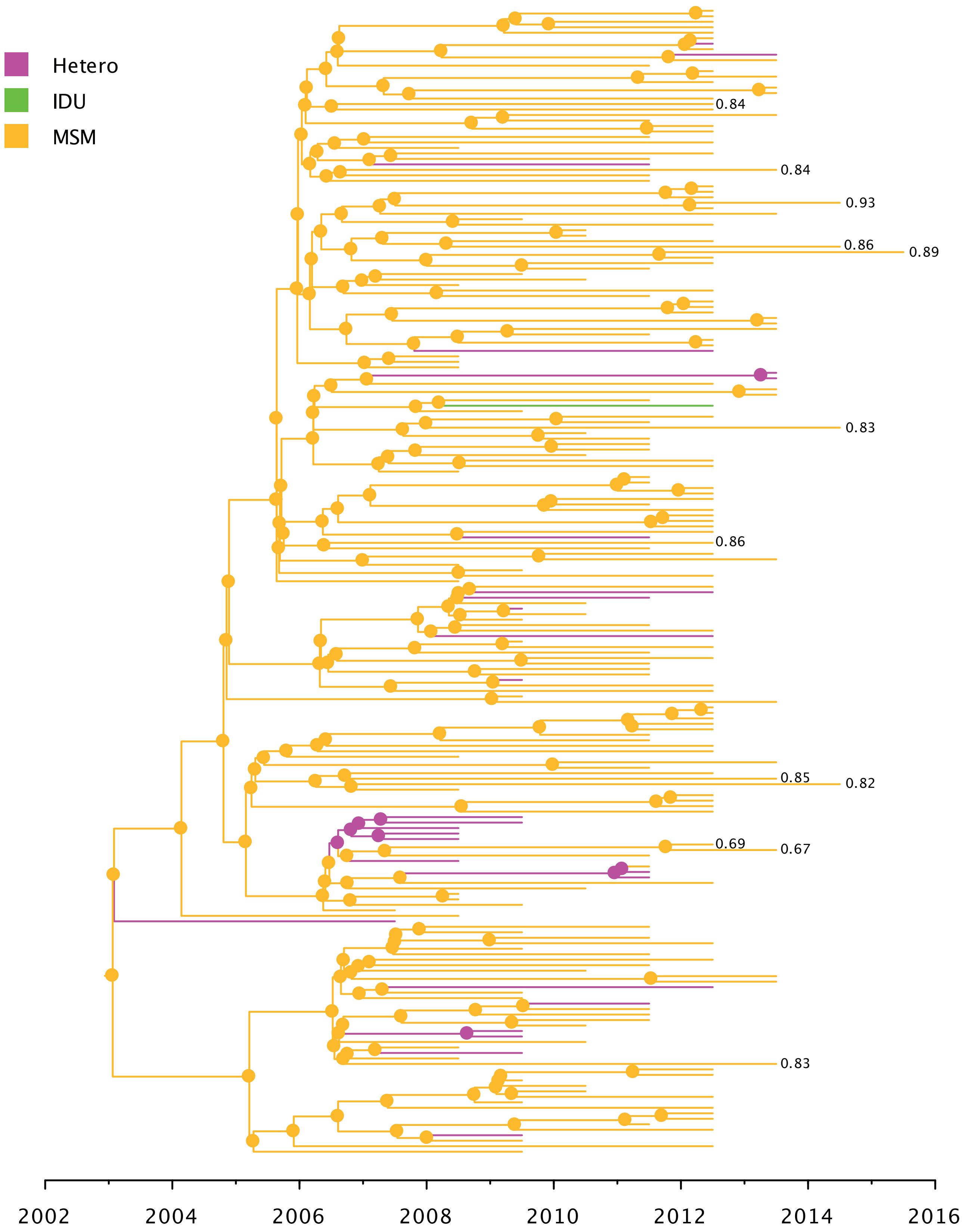

Supplementary Figure S4. Maximum-clade-credibility tree estimated from partial pol gene sequences of HIV-1 CRF55_01B.

Nodes are color coded by the most probable risk group of the descendent branches. The estimated of the most posterior probability of risk group for each sequence with unknown risk are shown. Color-coded risk groups are shown on the top left.

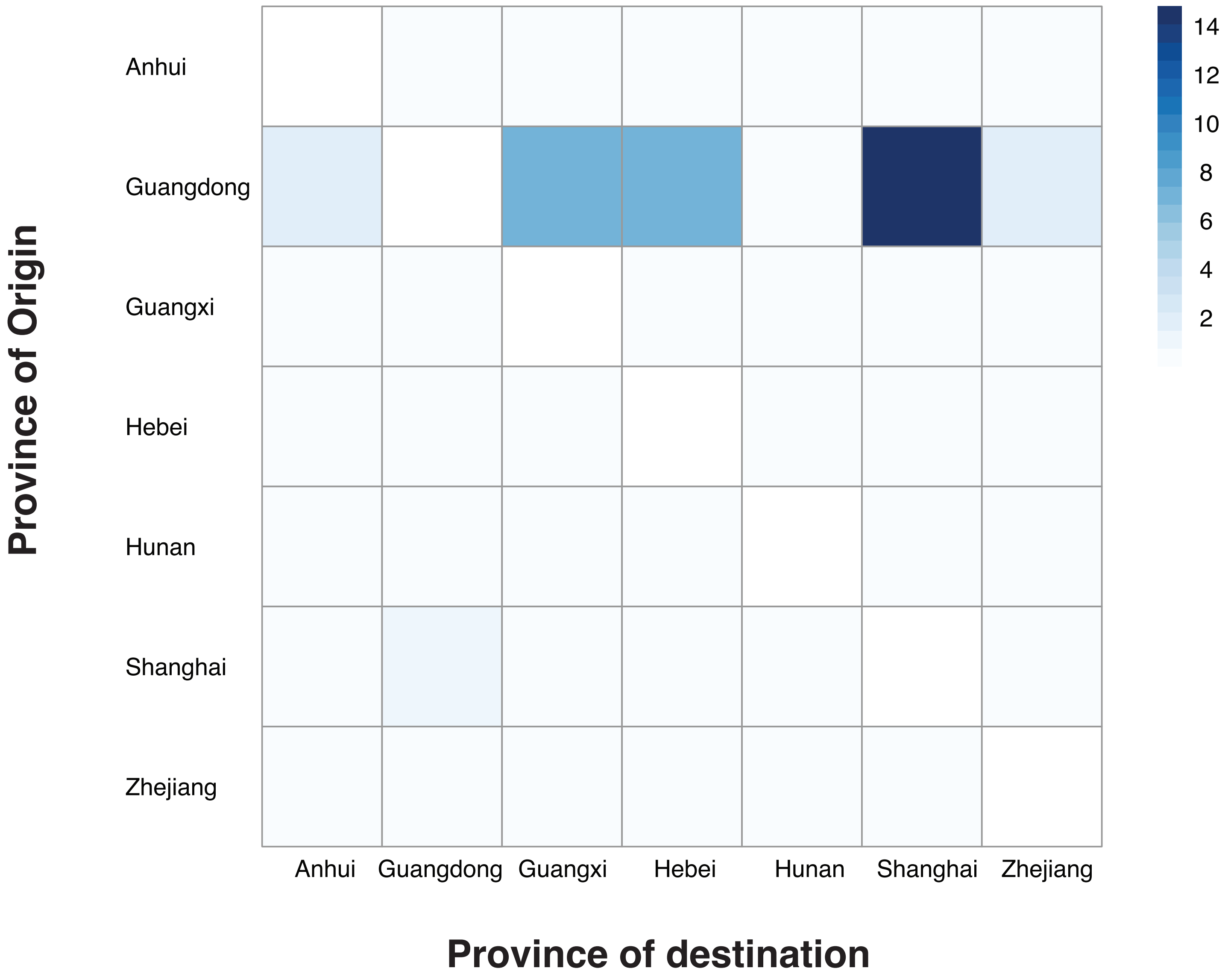

Supplementary Figure S5. Estimated migration events between each pair of geographic locations for HIV-1 CRF55_01B.

An increase in blueness represents a stronger movement signal. Transition direction is from the row trait (province of origin or seeding province) to the column trait (province of destination or receiving province). Posterior mean of jumps between each pair of geographic locations was obtained using a robust counting approach (see Materials and Methods).

Supplementary Table S1. HIV-1 CRF55_01B sequences used in the present study.

Acc. No.	Sequence name	Geographic source	Sampling year	Risk factor[a]
KF857359	LS411	Guangdong	2007	Hetero
KF857358	LS502	Guangdong	2007	MSM
KF857370	2008383	Guangdong	2008	MSM
KF857360	2008319	Guangdong	2008	MSM
KF857377	LS1005	Guangdong	2008	MSM
KF857361	LS1000	Guangdong	2008	MSM
KF857362	LS993	Guangdong	2008	MSM
KF857374	LS465	Guangdong	2008	MSM
KF857376	LS976	Guangdong	2008	MSM
KF857375	LS828	Guangdong	2008	MSM
KF857378	LS562	Guangdong	2008	MSM
KF857371	LS850	Guangdong	2008	MSM
KF857372	LS571	Guangdong	2008	MSM
KF857373	2008415	Guangdong	2008	MSM
KF857363	LS446	Guangdong	2008	MSM
KF857369	2008370	Guangdong	2008	MSM
KF857368	2008261	Guangdong	2008	MSM
KF857366	LS610	Guangdong	2008	Hetero
KF857367	LS434	Guangdong	2008	Hetero
KF857364	LS1010	Guangdong	2008	Hetero
KF857365	LS1011	Guangdong	2008	Hetero
KF857398	LS1373	Guangdong	2009	Hetero
KF857384	LS1279	Guangdong	2009	MSM
KF857386	LS1309	Guangdong	2009	MSM
KF857387	LS1305	Guangdong	2009	MSM
KF857393	LS1769	Guangdong	2009	MSM
KF857390	LS1666	Guangdong	2009	MSM
KF857381	LS1194	Guangdong	2009	MSM
KF857391	LS1560	Guangdong	2009	MSM
KF857399	LS1582	Guangdong	2009	Hetero
KF857400	LS1583	Guangdong	2009	Hetero
KF857380	LS1105	Guangdong	2009	MSM
KF857382	LS1231	Guangdong	2009	MSM
KF857379	LS1023	Guangdong	2009	MSM
KF857401	LS1665	Guangdong	2009	Hetero
KF857394	LS1618	Guangdong	2009	MSM
KF857388	LS1548	Guangdong	2009	MSM
KF857396	LS1026	Guangdong	2009	Hetero
KF857392	LS1673	Guangdong	2009	MSM
KF857389	LS1620	Guangdong	2009	MSM
KF857383	LS1243	Guangdong	2009	MSM
KF857395	LS1162	Guangdong	2009	Hetero
KF857385	LS1322	Guangdong	2009	MSM
KF857397	LS1236	Guangdong	2009	Hetero
JQ302616	2113	Guangxi	2009	Hetero

KF857410	LS2647	Guangdong	2010	MSM
KT379044	LS2326	Guangdong	2010	MSM
KF857411	LS2777	Guangdong	2010	MSM
KT379105	LS2796	Guangdong	2010	MSM
JX574661	HNCS102056	Hunan	2010	MSM
KF857405	LS2962	Guangdong	2010	MSM
KF857403	LS2663	Guangdong	2010	MSM
KF857404	LS2918	Guangdong	2010	MSM
KF857406	LS2515	Guangdong	2010	MSM
KT379131	LS3021	Guangdong	2010	MSM
KF857408	LS2656	Guangdong	2010	MSM
KF857407	LS2086	Guangdong	2010	MSM
KF857409	LS2497	Guangdong	2010	MSM
KF857419	LS3956	Guangdong	2011	MSM
KF857430	LS3923	Guangdong	2011	MSM
KT379357	LS4525	Guangdong	2011	Hetero
KF857431	LS3900	Guangdong	2011	MSM
KF857415	LS3559	Guangdong	2011	MSM
KT379343	LS4375	Guangdong	2011	MSM
KR187672	SF111340	Shanghai	2011	MSM
KF857423	LS3443	Guangdong	2011	MSM
KT379175	LS3353	Guangdong	2011	MSM
KT379194	LS3453	Guangdong	2011	MSM
KT379237	LS3730	Guangdong	2011	Hetero
KF857418	LS3925	Guangdong	2011	MSM
KF857427	LS3641	Guangdong	2011	MSM
KF857420	LS3114	Guangdong	2011	MSM
KF857424	LS3245	Guangdong	2011	MSM
KF857429	LS3508	Guangdong	2011	MSM
KT379277	LS3970	Guangdong	2011	Hetero
KF857432	LS4106	Guangdong	2011	MSM
KR187514	SF110032	Shanghai	2011	MSM
KT379274	LS3962	Guangdong	2011	MSM
KF927150	11HNCS500434	Hunan	2011	MSM
KF927151	11CN.GDDG325	Guangdong	2011	MSM
KF857413	LS3428	Guangdong	2011	MSM
KF857422	LS3240	Guangdong	2011	MSM
KF857412	LS3425	Guangdong	2011	MSM
KR187534	SF110202	Shanghai	2011	MSM
KT379148	LS3185	Guangdong	2011	MSM
KC183775	ANHUI_BB77Frag1	Anhui	2011	MSM
KF857426	LS3540	Guangdong	2011	MSM
KF857425	LS3401	Guangdong	2011	MSM
KF857417	LS3787	Guangdong	2011	MSM
JX574662	GDDG318	Guangdong	2011	MSM
JX574663	GDDG095	Guangdong	2011	MSM
KC183777	ANHUI_FY64	Anhui	2011	MSM
KT379356	LS4521	Guangdong	2011	Hetero

KF857414	LS3558	Guangdong	2011	MSM
KF857416	LS3784	Guangdong	2011	MSM
KF857421	LS3180	Guangdong	2011	MSM
KT379258	LS3864	Guangdong	2011	MSM
KF857428	LS3624	Guangdong	2011	MSM
KT379212	LS3561	Guangdong	2011	Hetero
KT379169	LS3317	Guangdong	2011	Hetero
KF857441	2012626	Guangdong	2012	MSM
KF857440	LS5410	Guangdong	2012	Hetero
KF857442	LS5463	Guangdong	2012	Hetero
KT379406	LS4747	Guangdong	2012	MSM
KT379758	LS6021	Guangdong	2012	MSM
KF857451	LS5369	Guangdong	2012	MSM
KT379460	LS4930	Guangdong	2012	MSM
KT379559	LS5299	Guangdong	2012	MSM
KT379455	LS4911	Guangdong	2012	MSM
KT379755	LS6010	Guangdong	2012	MSM
KF857456	LS5586	Guangdong	2012	MSM
KT379590	LS5417	Guangdong	2012	MSM
KF857447	LS5147	Guangdong	2012	MSM
KT379696	LS5792	Guangdong	2012	MSM
KT379851	LS6448	Guangdong	2012	MSM
KT379526	LS5158	Guangdong	2012	MSM
KT379523	LS5148	Guangdong	2012	Hetero
KT379451	LS4874	Guangdong	2012	MSM
KT379487	LS5045	Guangdong	2012	MSM
KT379463	LS4953	Guangdong	2012	MSM
KT379691	LS5774	Guangdong	2012	MSM
KT378720	H12053	Guangdong	2012	IDU
KT379784	LS6117	Guangdong	2012	MSM
KF857455	LS5576	Guangdong	2012	MSM
KF857450	LS5327	Guangdong	2012	MSM
KT379682	LS5730	Guangdong	2012	MSM
KT379478	LS5022	Guangdong	2012	MSM
KR187889	SF121497	Shanghai	2012	MSM
KT379515	LS5131	Guangdong	2012	MSM
KT379415	LS4769	Guangdong	2012	Hetero
KF857453	LS5431	Guangdong	2012	MSM
KF857452	LS5448	Guangdong	2012	MSM
KT379711	LS5840	Guangdong	2012	MSM
KT379556	LS5287	Guangdong	2012	MSM
KT379572	LS5339	Guangdong	2012	MSM
KT379725	LS5884	Guangdong	2012	MSM
KT379795	LS6179	Guangdong	2012	MSM
KF857454	LS5497	Guangdong	2012	MSM
KF857437	LS4661	Guangdong	2012	MSM
KY226139	GXME250	Guangxi	2012	n/a
KT379698	LS5794	Guangdong	2012	MSM

KR188002	SF121936	Shanghai	2012	MSM
KF857444	1936	Guangdong	2012	MSM
KR188003	SF121937	Shanghai	2012	MSM
KF857459	LS5705	Guangdong	2012	MSM
KT379753	LS6002	Guangdong	2012	MSM
KY226206	GXME373	Guangxi	2012	n/a
KR187823	SF121124	Shanghai	2012	MSM
KF857458	LS5701	Guangdong	2012	MSM
KT379642	LS5583	Guangdong	2012	MSM
KF857448	LS5231	Guangdong	2012	MSM
KT379861	LS6508	Guangdong	2012	MSM
KT379564	LS5312	Guangdong	2012	MSM
KT379779	LS6085	Guangdong	2012	MSM
KT379787	LS6127	Guangdong	2012	MSM
KT379720	LS5865	Guangdong	2012	MSM
KT379782	LS6100	Guangdong	2012	MSM
KT379561	LS5307	Guangdong	2012	Hetero
KT379728	LS5904	Guangdong	2012	MSM
KT379717	LS5854	Guangdong	2012	MSM
KT379663	LS5677	Guangdong	2012	MSM
KT379689	LS5749	Guangdong	2012	MSM
KF857457	LS5651	Guangdong	2012	MSM
KT379772	LS6061	Guangdong	2012	MSM
KT379450	LS4871	Guangdong	2012	MSM
KF857438	LS4726	Guangdong	2012	MSM
KT379453	LS4896	Guangdong	2012	MSM
KR187879	SF121426	Shanghai	2012	MSM
KF857445	1518	Guangdong	2012	MSM
KR187893	SF121518	Shanghai	2012	MSM
KT379668	LS5685	Guangdong	2012	MSM
KT379477	LS5017	Guangdong	2012	MSM
KT379508	LS5103	Guangdong	2012	MSM
KT379496	LS5061	Guangdong	2012	MSM
KF857460	LS5601	Guangdong	2012	MSM
KT379495	LS5060	Guangdong	2012	MSM
KF857449	LS5234	Guangdong	2012	MSM
KT379796	LS6181	Guangdong	2012	MSM
KY226207	GXME374	Guangxi	2012	n/a
KT379571	LS5333	Guangdong	2012	MSM
KF857439	LS5169	Guangdong	2012	MSM
KR188179	SF13G049	Shanghai	2013	MSM
KY226044	GXME075	Guangxi	2013	n/a
KR188134	SF131876	Shanghai	2013	MSM
KX198572	13CZ095	Hebei	2013	MSM
MF684332	12653	Hebei	2013	MSM
KM258826	HZ130991	Zhejiang	2013	MSM
KM258825	HZ130990	Zhejiang	2013	Hetero
MF684333	12739	Hebei	2013	MSM

KX198581	13TS1101	Hebei	2013	MSM
KR188267	SF13G208	Shanghai	2013	MSM
KF857446	130334	Guangdong	2013	MSM
KX198570	13CZ090	Hebei	2013	Hetero
MF684331	12650	Hebei	2013	Hetero
KF857443	946	Guangdong	2013	MSM
KR188102	SF130946	Shanghai	2013	MSM
KR188394	SF13G433	Shanghai	2013	MSM
KY226069	GXME125	Guangxi	2013	n/a
KR188423	SF13G491	Shanghai	2013	MSM
KY226194	GXME353	Guangxi	2013	n/a
KR188393	SF13G432	Shanghai	2013	MSM
KR188051	SF130300	Shanghai	2013	MSM
KR188258	SF13G190	Shanghai	2013	MSM
KY226226	GXME404	Guangxi	2013	n/a
MH010677	ZJ1412	Zhejiang	2014	n/a
MG706542	SJZ14108	Hebei	2014	n/a
MG706600	SJZ14176	Hebei	2014	n/a
MG706510	SJZ14074	Hebei	2014	n/a
MG706773	SJZ15110	Hebei	2015	n/a

[a]Risk factor: Hetero, heterosexual; IDU, injecting drug user; MSM, men having sex with men; n/a, not available.

Supplementary Table S2. List of HIV-1 CRF55_01B transmission clusters identified in the present study.

Acc. No.	Sequence name	Geographic source	Sampling year	Risk factor[a]	ClusterID
KT379755	LS6010	Guangdong	2012	MSM	1
KF857390	LS1666	Guangdong	2009	MSM	1
KF857392	LS1673	Guangdong	2009	MSM	2
KT379478	LS5022	Guangdong	2012	MSM	3
KT379784	LS6117	Guangdong	2012	MSM	4
KF857407	LS2086	Guangdong	2010	MSM	2
KT379451	LS4874	Guangdong	2012	MSM	5
KF857413	LS3428	Guangdong	2011	MSM	6
KR188003	SF121937	Shanghai	2012	MSM	7
KT379556	LS5287	Guangdong	2012	MSM	8
KT379044	LS2326	Guangdong	2010	MSM	9
KF857373	2008415	Guangdong	2008	MSM	10
KF857404	LS2918	Guangdong	2010	MSM	10
KT379642	LS5583	Guangdong	2012	MSM	11
KF857420	LS3114	Guangdong	2011	MSM	1
KF857411	LS2777	Guangdong	2010	MSM	12
KF857398	LS1373	Guangdong	2009	Hetero	9
KF857379	LS1023	Guangdong	2009	MSM	10
KF857460	LS5601	Guangdong	2012	MSM	13
KF857376	LS976	Guangdong	2008	MSM	1
KF857384	LS1279	Guangdong	2009	MSM	9
KF857389	LS1620	Guangdong	2009	MSM	2
KF857443	946	Guangdong	2013	MSM	14
KT379212	LS3561	Guangdong	2011	Hetero	15
KT379169	LS3317	Guangdong	2011	Hetero	15
KF857368	2008261	Guangdong	2008	MSM	16
KT379861	LS6508	Guangdong	2012	MSM	11
KT379105	LS2796	Guangdong	2010	MSM	12
KX198572	13CZ095	Hebei	2013	MSM	17
KF857438	LS4726	Guangdong	2012	MSM	2
KF857382	LS1231	Guangdong	2009	MSM	10
KT379194	LS3453	Guangdong	2011	MSM	1
KT379572	LS5339	Guangdong	2012	MSM	8
KT379495	LS5060	Guangdong	2012	MSM	13
KT379787	LS6127	Guangdong	2012	MSM	18
KF857427	LS3641	Guangdong	2011	MSM	1
KF857360	2008319	Guangdong	2008	MSM	1
KX198581	13TS1101	Hebei	2013	MSM	19
KF857444	1936	Guangdong	2012	MSM	7
KF857448	LS5231	Guangdong	2012	MSM	11
KT379772	LS6061	Guangdong	2012	MSM	2

KF857361	LS1000	Guangdong	2008	MSM	1
KT379753	LS6002	Guangdong	2012	MSM	20
KR188134	SF131876	Shanghai	2013	MSM	21
KF857429	LS3508	Guangdong	2011	MSM	4
MF684332	12653	Hebei	2013	MSM	17
KF857396	LS1026	Guangdong	2009	Hetero	22
KT379477	LS5017	Guangdong	2012	MSM	13
KF857424	LS3245	Guangdong	2011	MSM	4
KX198570	13CZ090	Hebei	2013	Hetero	23
KF857441	2012626	Guangdong	2012	MSM	9
KF857366	LS610	Guangdong	2008	Hetero	16
KF857447	LS5147	Guangdong	2012	MSM	24
KF857391	LS1560	Guangdong	2009	MSM	10
KF857414	LS3558	Guangdong	2011	MSM	25
MG706510	SJZ14074	Hebei	2014	nanna	20
KT379779	LS6085	Guangdong	2012	MSM	18
KT379590	LS5417	Guangdong	2012	MSM	24
KF857453	LS5431	Guangdong	2012	MSM	26
KT379663	LS5677	Guangdong	2012	MSM	25
KF857423	LS3443	Guangdong	2011	MSM	1
MF684333	12739	Hebei	2013	MSM	19
KT379523	LS5148	Guangdong	2012	Hetero	5
KF857381	LS1194	Guangdong	2009	MSM	1
KF857358	LS502	Guangdong	2007	MSM	16
KR187879	SF121426	Shanghai	2012	MSM	27
KR187893	SF121518	Shanghai	2012	MSM	27
KF857372	LS571	Guangdong	2008	MSM	10
KF857445	1518	Guangdong	2012	MSM	27
KT379795	LS6179	Guangdong	2012	MSM	1
KF857380	LS1105	Guangdong	2009	MSM	10
KF857418	LS3925	Guangdong	2011	MSM	1
KF857397	LS1236	Guangdong	2009	Hetero	16
KF857375	LS828	Guangdong	2008	MSM	1
KF857455	LS5576	Guangdong	2012	MSM	4
KF857388	LS1548	Guangdong	2009	MSM	22
KF857362	LS993	Guangdong	2008	MSM	1
KF857364	LS1010	Guangdong	2008	Hetero	16
KF857369	2008370	Guangdong	2008	MSM	16
KF857401	LS1665	Guangdong	2009	Hetero	10
KF857428	LS3624	Guangdong	2011	MSM	15
KT379460	LS4930	Guangdong	2012	MSM	16
KT379689	LS5749	Guangdong	2012	MSM	25
KF857395	LS1162	Guangdong	2009	Hetero	16
KF857393	LS1769	Guangdong	2009	MSM	1
KT379496	LS5061	Guangdong	2012	MSM	13

KF857422	LS3240	Guangdong	2011	MSM	6
KF857410	LS2647	Guangdong	2010	MSM	9
KF857367	LS434	Guangdong	2008	Hetero	16
KR188002	SF121936	Shanghai	2012	MSM	7
KF857365	LS1011	Guangdong	2008	Hetero	16
KT379526	LS5158	Guangdong	2012	MSM	5
KF857408	LS2656	Guangdong	2010	MSM	2
MF684331	12650	Hebei	2013	Hetero	23
KR188102	SF130946	Shanghai	2013	MSM	14
KF857452	LS5448	Guangdong	2012	MSM	26
KT379559	LS5299	Guangdong	2012	MSM	21
KT379682	LS5730	Guangdong	2012	MSM	3

[a]Risk factor: Hetero, heterosexual; MSM, men having sex with men.

Supplementary Table S3. Characteristics of HIV-1 CRF55_01B transmission clusters identified in the present study.

		Province				Risk group		
**Cluster No.**	**Cluster size**	**Guangdong**	**Hebei**	**Shanghai**	**Sampling period**	**Hetero**	**MSM**	**n/a**
1	15	15			2008-2012		15	
2	6	6			2009-2012		6	
3	2	2			2012		2	
4	4	4			2011-2012		4	
5	3	3			2012	1	2	
6	2	2			2011		2	
7	3	1		2	2012		3	
8	2	2			2012		2	
9	5	5			2009-2012	1	4	
10	8	8			2008-2010	1	7	
11	3	3			2012		3	
12	2	2			2010		2	
13	4	4			2012		4	
14	2	1		1	2013		2	
15	3	3			2011	2	1	
16	10	10			2007-2012	6	4	
17	2		2		2013		2	
18	2	2			2012		2	
19	2		2		2013		2	
20	2	1	1		2012-2014		1	1
21	2	1		1	2012-2013		2	
22	2	2			2009	1	1	
23	2		2		2013	2		
24	2	2			2012		2	
25	3	3			2011-2012		3	
26	2	2			2012		2	
27	3	1		2	2012		3	
**Total**	**98**	**85**	**7**	**6**	**2007-2014**	**14**	**83**	**1**

[a]Risk factor: Hetero, heterosexual; MSM, men having sex with men; n/a, not available.
